# Supplementary material for: Sudachitin and Nobiletin Stimulate Lipolysis via Activation of the cAMP/PKA/HSL Pathway in 3T3-L1 Adipocytes
Source: Foods. 2023 May 10;12(10):1947. doi: 10.3390/foods12101947 (PMC10217623; doi:10.3390/foods12101947)
Supplement: Supplementary file 1 [file foods-12-01947-s001.zip › foods-2364921-supplementary.pdf]

Supplementary information

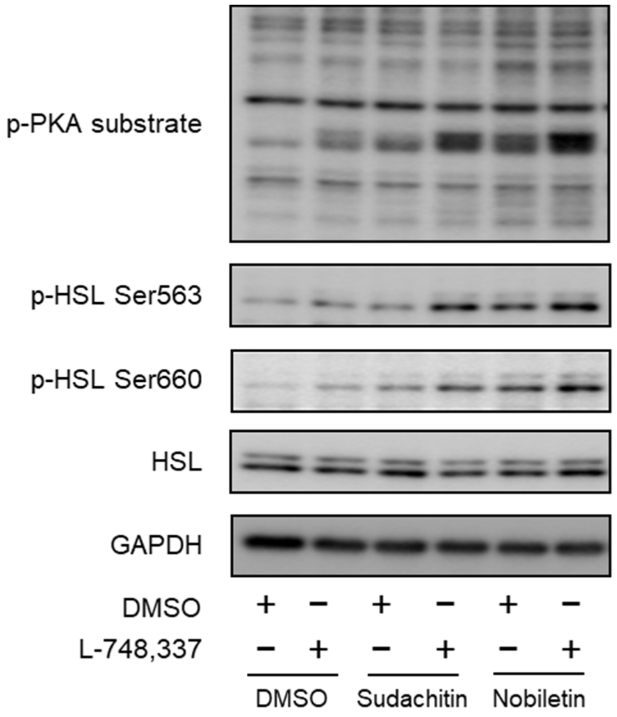

Supplementary Figure S1. Effects of β3-adrenergic receptor-selective antagonist on sudachitin- and nobiletin-induced phosphorylation of the PKA substrate and HSL. Protein expression of PKA substrate and HSL phosphorylated at Ser563, HSL phosphorylated at Ser660, total HSL, and GAPDH. 3T3-L1 adipocytes are treated with DMSO (0.5 μL/mL) or L-748,337 (20 μM) and before treatment with DMSO (0.5 μL/mL), sudachitin or nobiletin (30 μM). DMSO, dimethyl sulfoxide; GAPDH, glyceraldehyde 3-phosphate dehydrogenase; HSL, hormone-sensitive lipase; PKA, protein kinase A.
